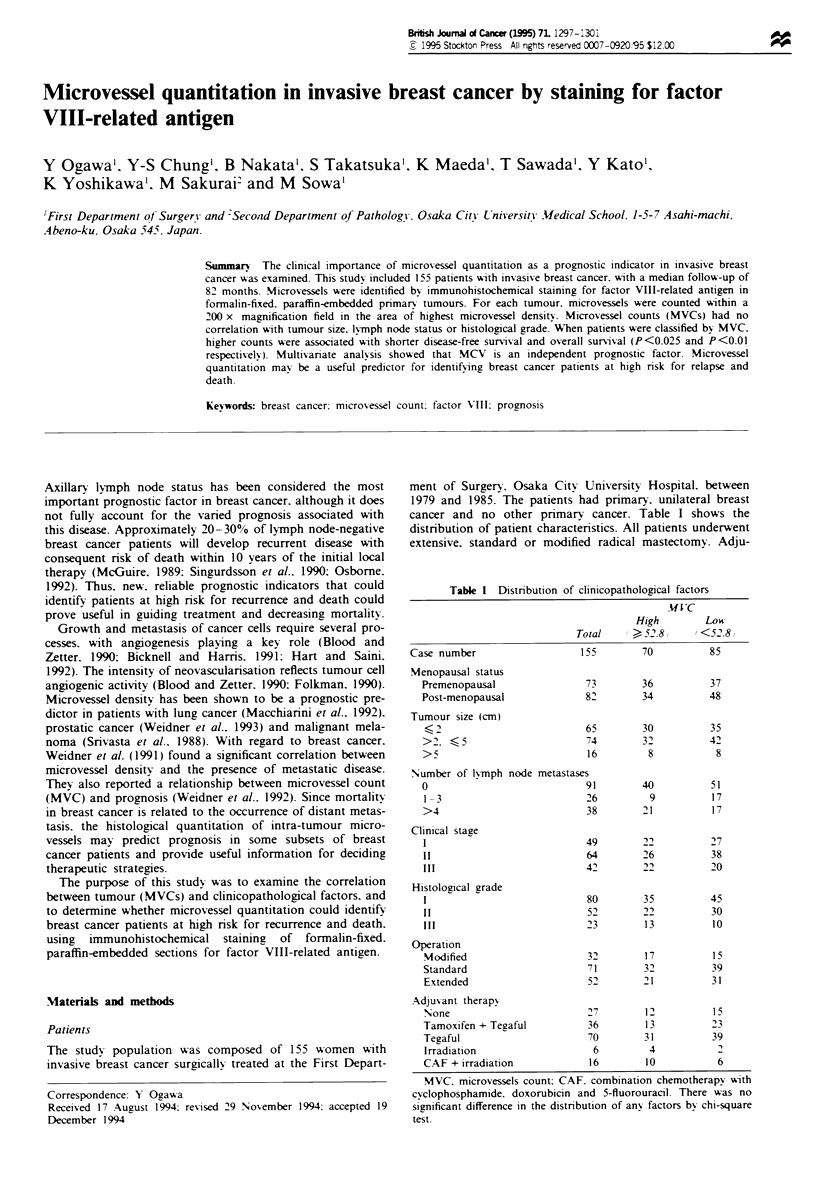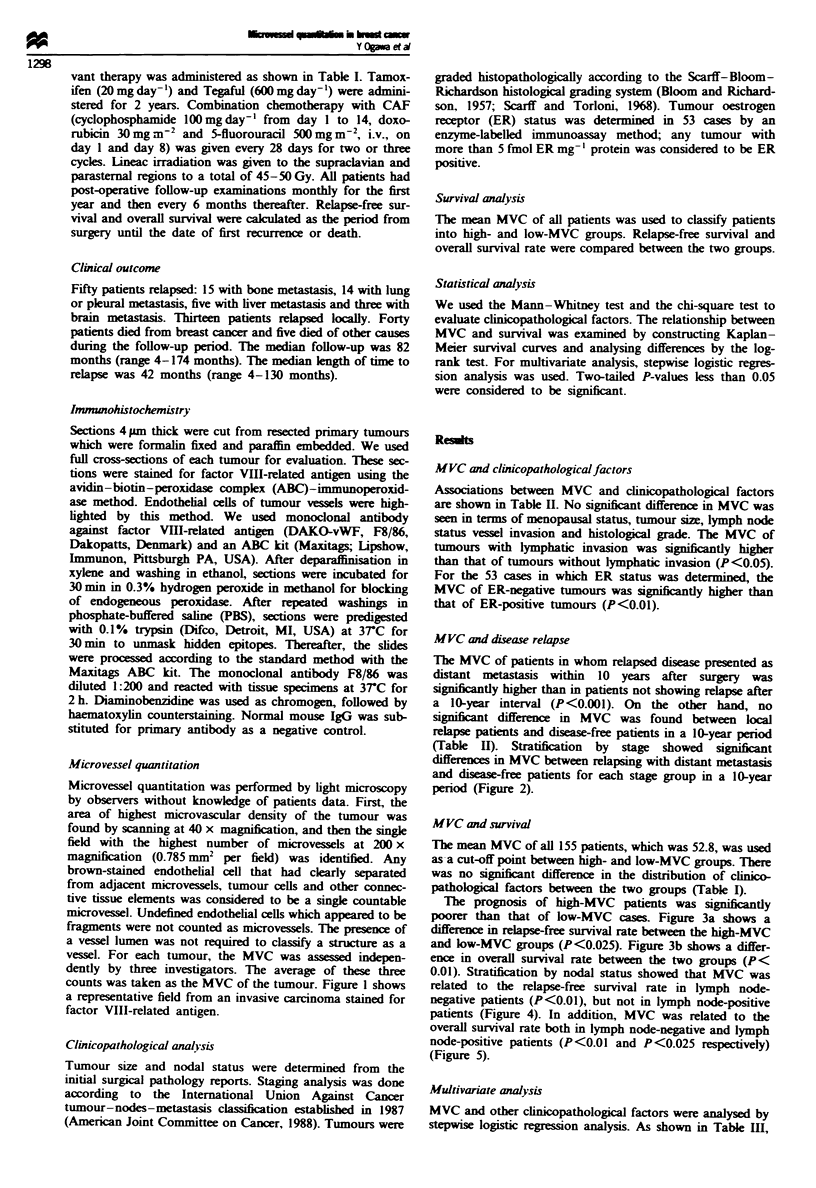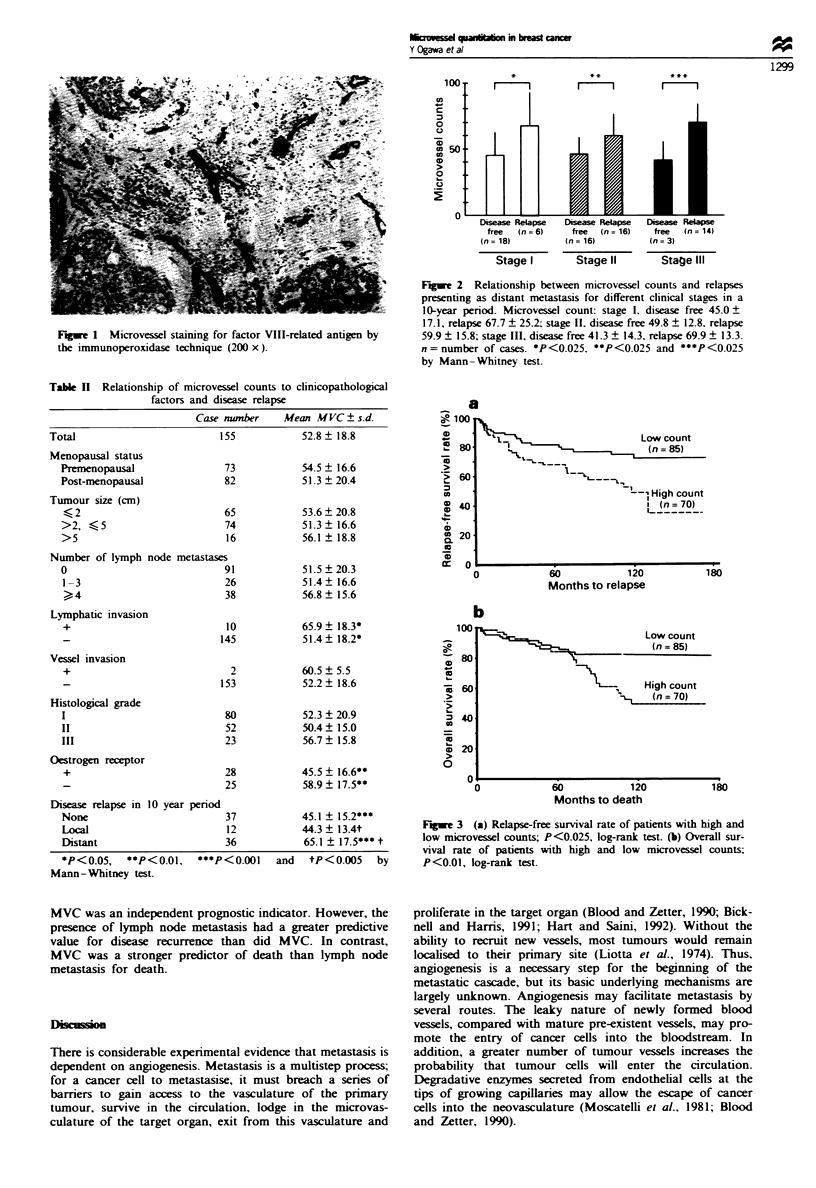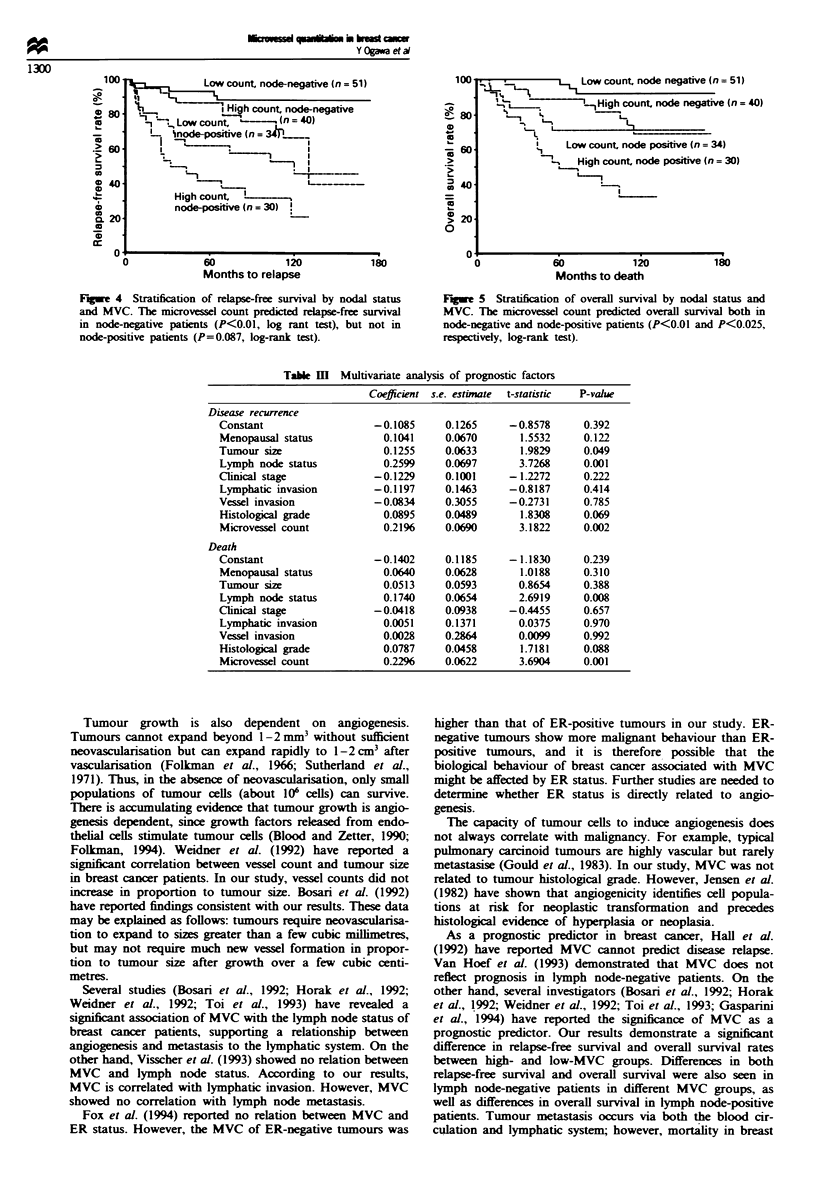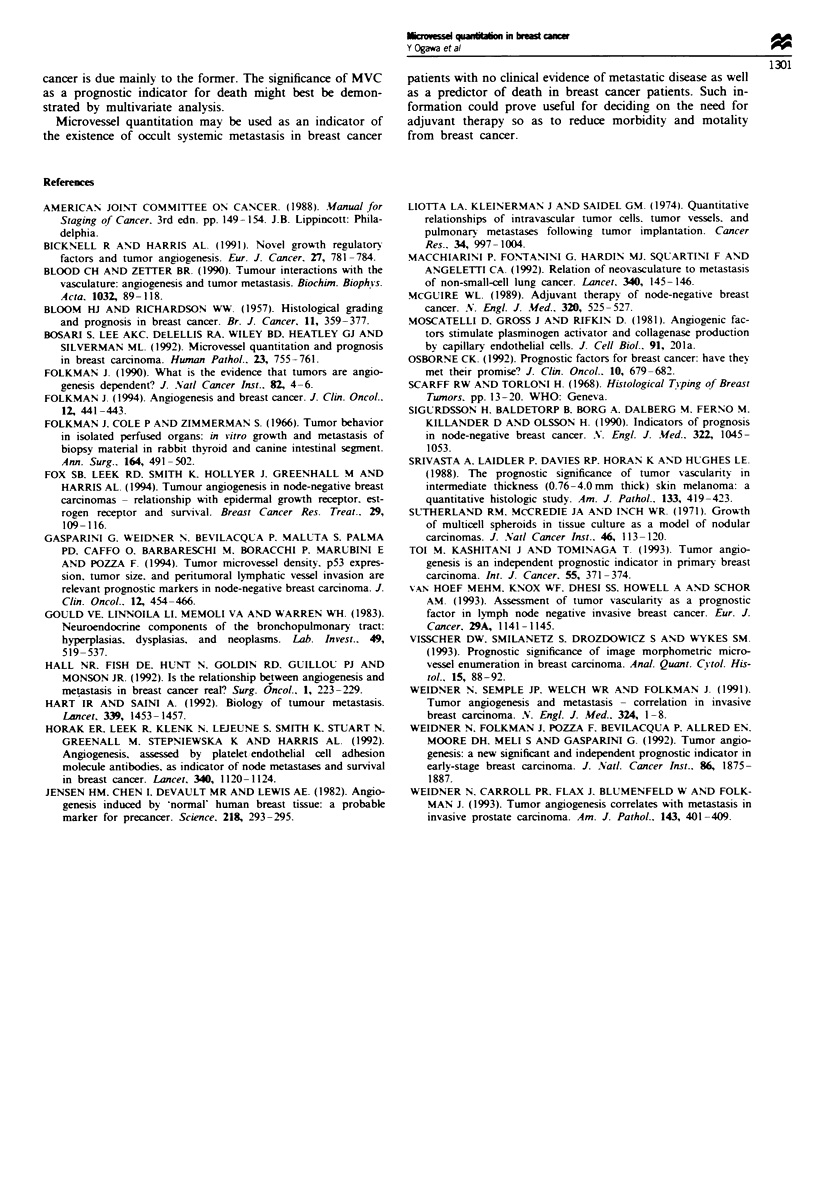# Microvessel quantitation in invasive breast cancer by staining for factor VIII-related antigen.

**DOI:** 10.1038/bjc.1995.251

**Published:** 1995-06

**Authors:** Y. Ogawa, Y. S. Chung, B. Nakata, S. Takatsuka, K. Maeda, T. Sawada, Y. Kato, K. Yoshikawa, M. Sakurai, M. Sowa

**Affiliations:** First Department of Surgery, Osaka City University Medical School, Japan.

## Abstract

**Images:**


British Journal of Cancer (1995) 71, 1297-1301

© 1995 Stockton Press All rights reserved 0007-0920/95 $12.00

### Microvessel quantitation in invasive breast cancer by staining for factor VIII-related antigen

Y Ogawa[1], Y-S Chung[1], B Nakata[1], S Takatsuka[1], K Maeda[1], T Sawada[1], Y Kato[1], K Yoshikawa[1], M Sakurai[2] and M Sowa[1]

[1]*First Department of Surgery and* [2]*Second Department of Pathology, Osaka City University Medical School, 1-5-7 Asahi-machi, Abeno-ku, Osaka 545, Japan.*

**Summary** The clinical importance of microvessel quantitation as a prognostic indicator in invasive breast cancer was examined. This study included 155 patients with invasive breast cancer, with a median follow-up of 82 months. Microvessels were identified by immunohistochemical staining for factor VIII-related antigen in formalin-fixed, paraffin-embedded primary tumours. For each tumour, microvessels were counted within a 200 × magnification field in the area of highest microvessel density. Microvessel counts (MVCs) had no correlation with tumour size, lymph node status or histological grade. When patients were classified by MVC, higher counts were associated with shorter disease-free survival and overall survival ($P<0.025$ and $P<0.01$ respectively). Multivariate analysis showed that MCV is an independent prognostic factor. Microvessel quantitation may be a useful predictor for identifying breast cancer patients at high risk for relapse and death.

**Keywords:** breast cancer; microvessel count; factor VIII; prognosis

Axillary lymph node status has been considered the most important prognostic factor in breast cancer, although it does not fully account for the varied prognosis associated with this disease. Approximately 20–30% of lymph node-negative breast cancer patients will develop recurrent disease with consequent risk of death within 10 years of the initial local therapy (McGuire, 1989; Singurdsson *et al.*, 1990; Osborne, 1992). Thus, new, reliable prognostic indicators that could identify patients at high risk for recurrence and death could prove useful in guiding treatment and decreasing mortality.

Growth and metastasis of cancer cells require several processes, with angiogenesis playing a key role (Blood and Zetter, 1990; Bicknell and Harris, 1991; Hart and Saini, 1992). The intensity of neovascularisation reflects tumour cell angiogenic activity (Blood and Zetter, 1990; Folkman, 1990). Microvessel density has been shown to be a prognostic predictor in patients with lung cancer (Macchiarini *et al.*, 1992), prostatic cancer (Weidner *et al.*, 1993) and malignant melanoma (Srivasta *et al.*, 1988). With regard to breast cancer, Weidner *et al.* (1991) found a significant correlation between microvessel density and the presence of metastatic disease. They also reported a relationship between microvessel count (MVC) and prognosis (Weidner *et al.*, 1992). Since mortality in breast cancer is related to the occurrence of distant metastasis, the histological quantitation of intra-tumour microvessels may predict prognosis in some subsets of breast cancer patients and provide useful information for deciding therapeutic strategies.

The purpose of this study was to examine the correlation between tumour (MVCs) and clinicopathological factors, and to determine whether microvessel quantitation could identify breast cancer patients at high risk for recurrence and death, using immunohistochemical staining of formalin-fixed, paraffin-embedded sections for factor VIII-related antigen.

### Materials and methods

### *Patients*

The study population was composed of 155 women with invasive breast cancer surgically treated at the First Department of Surgery, Osaka City University Hospital, between 1979 and 1985. The patients had primary, unilateral breast cancer and no other primary cancer. Table I shows the distribution of patient characteristics. All patients underwent extensive, standard or modified radical mastectomy. Adju-

**Table I** Distribution of clinicopathological factors

| | | *MVC* | |
|---|---|---|---|
| | *Total* | *High* (≥52.8) | *Low* (<52.8) |
| Case number | 155 | 70 | 85 |
| Menopausal status | | | |
| Premenopausal | 73 | 36 | 37 |
| Post-menopausal | 82 | 34 | 48 |
| Tumour size (cm) | | | |
| ≤2 | 65 | 30 | 35 |
| >2, ≤5 | 74 | 32 | 42 |
| >5 | 16 | 8 | 8 |
| Number of lymph node metastases | | | |
| 0 | 91 | 40 | 51 |
| 1–3 | 26 | 9 | 17 |
| >4 | 38 | 21 | 17 |
| Clinical stage | | | |
| I | 49 | 22 | 27 |
| II | 64 | 26 | 38 |
| III | 42 | 22 | 20 |
| Histological grade | | | |
| I | 80 | 35 | 45 |
| II | 52 | 22 | 30 |
| III | 23 | 13 | 10 |
| Operation | | | |
| Modified | 32 | 17 | 15 |
| Standard | 71 | 32 | 39 |
| Extended | 52 | 21 | 31 |
| Adjuvant therapy | | | |
| None | 27 | 12 | 15 |
| Tamoxifen + Tegaful | 36 | 13 | 23 |
| Tegaful | 70 | 31 | 39 |
| Irradiation | 6 | 4 | 2 |
| CAF + irradiation | 16 | 10 | 6 |

MVC, microvessels count; CAF, combination chemotherapy with cyclophosphamide, doxorubicin and 5-fluorouracil. There was no significant difference in the distribution of any factors by chi-square test.

Correspondence: Y Ogawa
Received 17 August 1994; revised 29 November 1994; accepted 19 December 1994

vant therapy was administered as shown in Table I. Tamoxifen (20 mg day$^{-1}$) and Tegaful (600 mg day$^{-1}$) were administered for 2 years. Combination chemotherapy with CAF (cyclophosphamide 100 mg day$^{-1}$ from day 1 to 14, doxorubicin 30 mg m$^{-2}$ and 5-fluorouracil 500 mg m$^{-2}$, i.v., on day 1 and day 8) was given every 28 days for two or three cycles. Lineac irradiation was given to the supraclavian and parasternal regions to a total of 45–50 Gy. All patients had post-operative follow-up examinations monthly for the first year and then every 6 months thereafter. Relapse-free survival and overall survival were calculated as the period from surgery until the date of first recurrence or death.

*Clinical outcome*

Fifty patients relapsed: 15 with bone metastasis, 14 with lung or pleural metastasis, five with liver metastasis and three with brain metastasis. Thirteen patients relapsed locally. Forty patients died from breast cancer and five died of other causes during the follow-up period. The median follow-up was 82 months (range 4–174 months). The median length of time to relapse was 42 months (range 4–130 months).

*Immunohistochemistry*

Sections 4 μm thick were cut from resected primary tumours which were formalin fixed and paraffin embedded. We used full cross-sections of each tumour for evaluation. These sections were stained for factor VIII-related antigen using the avidin–biotin–peroxidase complex (ABC)-immunoperoxidase method. Endothelial cells of tumour vessels were highlighted by this method. We used monoclonal antibody against factor VIII-related antigen (DAKO-vWF, F8/86, Dakopatts, Denmark) and an ABC kit (Maxitags; Lipshow, Immunon, Pittsburgh PA, USA). After deparaffinisation in xylene and washing in ethanol, sections were incubated for 30 min in 0.3% hydrogen peroxide in methanol for blocking of endogeneous peroxidase. After repeated washings in phosphate-buffered saline (PBS), sections were predigested with 0.1% trypsin (Difco, Detroit, MI, USA) at 37°C for 30 min to unmask hidden epitopes. Thereafter, the slides were processed according to the standard method with the Maxitags ABC kit. The monoclonal antibody F8/86 was diluted 1:200 and reacted with tissue specimens at 37°C for 2 h. Diaminobenzidine was used as chromogen, followed by haematoxylin counterstaining. Normal mouse IgG was substituted for primary antibody as a negative control.

*Microvessel quantitation*

Microvessel quantitation was performed by light microscopy by observers without knowledge of patients data. First, the area of highest microvascular density of the tumour was found by scanning at 40 × magnification, and then the single field with the highest number of microvessels at 200 × magnification (0.785 mm$^2$ per field) was identified. Any brown-stained endothelial cell that had clearly separated from adjacent microvessels, tumour cells and other connective tissue elements was considered to be a single countable microvessel. Undefined endothelial cells which appeared to be fragments were not counted as microvessels. The presence of a vessel lumen was not required to classify a structure as a vessel. For each tumour, the MVC was assessed independently by three investigators. The average of these three counts was taken as the MVC of the tumour. Figure 1 shows a representative field from an invasive carcinoma stained for factor VIII-related antigen.

*Clinicopathological analysis*

Tumour size and nodal status were determined from the initial surgical pathology reports. Staging analysis was done according to the International Union Against Cancer tumour–nodes–metastasis classification established in 1987 (American Joint Committee on Cancer, 1988). Tumours were graded histopathologically according to the Scarff–Bloom–Richardson histological grading system (Bloom and Richardson, 1957; Scarff and Torloni, 1968). Tumour oestrogen receptor (ER) status was determined in 53 cases by an enzyme-labelled immunoassay method; any tumour with more than 5 fmol ER mg$^{-1}$ protein was considered to be ER positive.

*Survival analysis*

The mean MVC of all patients was used to classify patients into high- and low-MVC groups. Relapse-free survival and overall survival rate were compared between the two groups.

*Statistical analysis*

We used the Mann–Whitney test and the chi-square test to evaluate clinicopathological factors. The relationship between MVC and survival was examined by constructing Kaplan–Meier survival curves and analysing differences by the log-rank test. For multivariate analysis, stepwise logistic regression analysis was used. Two-tailed $P$-values less than 0.05 were considered to be significant.

### Results

*MVC and clinicopathological factors*

Associations between MVC and clinicopathological factors are shown in Table II. No significant difference in MVC was seen in terms of menopausal status, tumour size, lymph node status vessel invasion and histological grade. The MVC of tumours with lymphatic invasion was significantly higher than that of tumours without lymphatic invasion ($P<0.05$). For the 53 cases in which ER status was determined, the MVC of ER-negative tumours was significantly higher than that of ER-positive tumours ($P<0.01$).

*MVC and disease relapse*

The MVC of patients in whom relapsed disease presented as distant metastasis within 10 years after surgery was significantly higher than in patients not showing relapse after a 10-year interval ($P<0.001$). On the other hand, no significant difference in MVC was found between local relapse patients and disease-free patients in a 10-year period (Table II). Stratification by stage showed significant differences in MVC between relapsing with distant metastasis and disease-free patients for each stage group in a 10-year period (Figure 2).

*MVC and survival*

The mean MVC of all 155 patients, which was 52.8, was used as a cut-off point between high- and low-MVC groups. There was no significant difference in the distribution of clinicopathological factors between the two groups (Table I).

The prognosis of high-MVC patients was significantly poorer than that of low-MVC cases. Figure 3a shows a difference in relapse-free survival rate between the high-MVC and low-MVC groups ($P<0.025$). Figure 3b shows a difference in overall survival rate between the two groups ($P<0.01$). Stratification by nodal status showed that MVC was related to the relapse-free survival rate in lymph node-negative patients ($P<0.01$), but not in lymph node-positive patients (Figure 4). In addition, MVC was related to the overall survival rate both in lymph node-negative and lymph node-positive patients ($P<0.01$ and $P<0.025$ respectively) (Figure 5).

*Multivariate analysis*

MVC and other clinicopathological factors were analysed by stepwise logistic regression analysis. As shown in Table III,

**Figure 1** Microvessel staining for factor VIII-related antigen by the immunoperoxidase technique (200 ×).

**Table II** Relationship of microvessel counts to clinicopathological factors and disease relapse

| | *Case number* | *Mean MVC ± s.d.* |
|---|---|---|
| Total | 155 | 52.8 ± 18.8 |
| Menopausal status | | |
| Premenopausal | 73 | 54.5 ± 16.6 |
| Post-menopausal | 82 | 51.3 ± 20.4 |
| Tumour size (cm) | | |
| ⩽2 | 65 | 53.6 ± 20.8 |
| >2, ⩽5 | 74 | 51.3 ± 16.6 |
| >5 | 16 | 56.1 ± 18.8 |
| Number of lymph node metastases | | |
| 0 | 91 | 51.5 ± 20.3 |
| 1–3 | 26 | 51.4 ± 16.6 |
| ⩾4 | 38 | 56.8 ± 15.6 |
| Lymphatic invasion | | |
| + | 10 | 65.9 ± 18.3* |
| – | 145 | 51.4 ± 18.2* |
| Vessel invasion | | |
| + | 2 | 60.5 ± 5.5 |
| – | 153 | 52.2 ± 18.6 |
| Histological grade | | |
| I | 80 | 52.3 ± 20.9 |
| II | 52 | 50.4 ± 15.0 |
| III | 23 | 56.7 ± 15.8 |
| Oestrogen receptor | | |
| + | 28 | 45.5 ± 16.6** |
| – | 25 | 58.9 ± 17.5** |
| Disease relapse in 10 year period | | |
| None | 37 | 45.1 ± 15.2*** |
| Local | 12 | 44.3 ± 13.4† |
| Distant | 36 | 65.1 ± 17.5*** † |

*$P<0.05$, **$P<0.01$, ***$P<0.001$ and †$P<0.005$ by Mann–Whitney test.

**Figure 2** Relationship between microvessel counts and relapses presenting as distant metastasis for different clinical stages in a 10-year period. Microvessel count: stage I, disease free 45.0 ± 17.1, relapse 67.7 ± 25.2; stage II, disease free 49.8 ± 12.8, relapse 59.9 ± 15.8; stage III, disease free 41.3 ± 14.3, relapse 69.9 ± 13.3. $n$ = number of cases. *$P<0.025$, **$P<0.025$ and ***$P<0.025$ by Mann–Whitney test.

**Figure 3** (**a**) Relapse-free survival rate of patients with high and low microvessel counts; $P<0.025$, log-rank test. (**b**) Overall survival rate of patients with high and low microvessel counts; $P<0.01$, log-rank test.

MVC was an independent prognostic indicator. However, the presence of lymph node metastasis had a greater predictive value for disease recurrence than did MVC. In contrast, MVC was a stronger predictor of death than lymph node metastasis for death.

### Discussion

There is considerable experimental evidence that metastasis is dependent on angiogenesis. Metastasis is a multistep process; for a cancer cell to metastasise, it must breach a series of barriers to gain access to the vasculature of the primary tumour, survive in the circulation, lodge in the microvasculature of the target organ, exit from this vasculature and proliferate in the target organ (Blood and Zetter, 1990; Bicknell and Harris, 1991; Hart and Saini, 1992). Without the ability to recruit new vessels, most tumours would remain localised to their primary site (Liotta *et al*., 1974). Thus, angiogenesis is a necessary step for the beginning of the metastatic cascade, but its basic underlying mechanisms are largely unknown. Angiogenesis may facilitate metastasis by several routes. The leaky nature of newly formed blood vessels, compared with mature pre-existent vessels, may promote the entry of cancer cells into the bloodstream. In addition, a greater number of tumour vessels increases the probability that tumour cells will enter the circulation. Degradative enzymes secreted from endothelial cells at the tips of growing capillaries may allow the escape of cancer cells into the neovasculature (Moscatelli *et al*., 1981; Blood and Zetter, 1990).

**Figure 4** Stratification of relapse-free survival by nodal status and MVC. The microvessel count predicted relapse-free survival in node-negative patients ($P<0.01$, log rant test), but not in node-positive patients ($P=0.087$, log-rank test).

**Figure 5** Stratification of overall survival by nodal status and MVC. The microvessel count predicted overall survival both in node-negative and node-positive patients ($P<0.01$ and $P<0.025$, respectively, log-rank test).

**Table III** Multivariate analysis of prognostic factors

| | *Coefficient* | *s.e. estimate* | t-*statistic* | P-*value* |
|---|---|---|---|---|
| *Disease recurrence* | | | | |
| Constant | −0.1085 | 0.1265 | −0.8578 | 0.392 |
| Menopausal status | 0.1041 | 0.0670 | 1.5532 | 0.122 |
| Tumour size | 0.1255 | 0.0633 | 1.9829 | 0.049 |
| Lymph node status | 0.2599 | 0.0697 | 3.7268 | 0.001 |
| Clinical stage | −0.1229 | 0.1001 | −1.2272 | 0.222 |
| Lymphatic invasion | −0.1197 | 0.1463 | −0.8187 | 0.414 |
| Vessel invasion | −0.0834 | 0.3055 | −0.2731 | 0.785 |
| Histological grade | 0.0895 | 0.0489 | 1.8308 | 0.069 |
| Microvessel count | 0.2196 | 0.0690 | 3.1822 | 0.002 |
| *Death* | | | | |
| Constant | −0.1402 | 0.1185 | −1.1830 | 0.239 |
| Menopausal status | 0.0640 | 0.0628 | 1.0188 | 0.310 |
| Tumour size | 0.0513 | 0.0593 | 0.8654 | 0.388 |
| Lymph node status | 0.1740 | 0.0654 | 2.6919 | 0.008 |
| Clinical stage | −0.0418 | 0.0938 | −0.4455 | 0.657 |
| Lymphatic invasion | 0.0051 | 0.1371 | 0.0375 | 0.970 |
| Vessel invasion | 0.0028 | 0.2864 | 0.0099 | 0.992 |
| Histological grade | 0.0787 | 0.0458 | 1.7181 | 0.088 |
| Microvessel count | 0.2296 | 0.0622 | 3.6904 | 0.001 |

Tumour growth is also dependent on angiogenesis. Tumours cannot expand beyond 1–2 $mm^3$ without sufficient neovascularisation but can expand rapidly to 1–2 $cm^3$ after vascularisation (Folkman *et al.*, 1966; Sutherland *et al.*, 1971). Thus, in the absence of neovascularisation, only small populations of tumour cells (about $10^6$ cells) can survive. There is accumulating evidence that tumour growth is angiogenesis dependent, since growth factors released from endothelial cells stimulate tumour cells (Blood and Zetter, 1990; Folkman, 1994). Weidner *et al.* (1992) have reported a significant correlation between vessel count and tumour size in breast cancer patients. In our study, vessel counts did not increase in proportion to tumour size. Bosari *et al.* (1992) have reported findings consistent with our results. These data may be explained as follows: tumours require neovascularisation to expand to sizes greater than a few cubic millimetres, but may not require much new vessel formation in proportion to tumour size after growth over a few cubic centimetres.

Several studies (Bosari *et al.*, 1992; Horak *et al.*, 1992; Weidner *et al.*, 1992; Toi *et al.*, 1993) have revealed a significant association of MVC with the lymph node status of breast cancer patients, supporting a relationship between angiogenesis and metastasis to the lymphatic system. On the other hand, Visscher *et al.* (1993) showed no relation between MVC and lymph node status. According to our results, MVC is correlated with lymphatic invasion. However, MVC showed no correlation with lymph node metastasis.

Fox *et al.* (1994) reported no relation between MVC and ER status. However, the MVC of ER-negative tumours was higher than that of ER-positive tumours in our study. ER-negative tumours show more malignant behaviour than ER-positive tumours, and it is therefore possible that the biological behaviour of breast cancer associated with MVC might be affected by ER status. Further studies are needed to determine whether ER status is directly related to angiogenesis.

The capacity of tumour cells to induce angiogenesis does not always correlate with malignancy. For example, typical pulmonary carcinoid tumours are highly vascular but rarely metastasise (Gould *et al.*, 1983). In our study, MVC was not related to tumour histological grade. However, Jensen *et al.* (1982) have shown that angiogenicity identifies cell populations at risk for neoplastic transformation and precedes histological evidence of hyperplasia or neoplasia.

As a prognostic predictor in breast cancer, Hall *et al.* (1992) have reported MVC cannot predict disease relapse. Van Hoef *et al.* (1993) demonstrated that MVC does not reflect prognosis in lymph node-negative patients. On the other hand, several investigators (Bosari *et al.*, 1992; Horak *et al.*, 1992; Weidner *et al.*, 1992; Toi *et al.*, 1993; Gasparini *et al.*, 1994) have reported the significance of MVC as a prognostic predictor. Our results demonstrate a significant difference in relapse-free survival and overall survival rates between high- and low-MVC groups. Differences in both relapse-free survival and overall survival were also seen in lymph node-negative patients in different MVC groups, as well as differences in overall survival in lymph node-positive patients. Tumour metastasis occurs via both the blood circulation and lymphatic system; however, mortality in breast cancer is due mainly to the former. The significance of MVC as a prognostic indicator for death might best be demonstrated by multivariate analysis.

Microvessel quantitation may be used as an indicator of the existence of occult systemic metastasis in breast cancer patients with no clinical evidence of metastatic disease as well as a predictor of death in breast cancer patients. Such information could prove useful for deciding on the need for adjuvant therapy so as to reduce morbidity and motality from breast cancer.

### References

AMERICAN JOINT COMMITTEE ON CANCER. (1988). *Manual for Staging of Cancer*. 3rd edn. pp. 149-154. J.B. Lippincott: Philadelphia.

BICKNELL R AND HARRIS AL. (1991). Novel growth regulatory factors and tumor angiogenesis. *Eur. J. Cancer*, **27**, 781-784.

BLOOD CH AND ZETTER BR. (1990). Tumour interactions with the vasculature: angiogenesis and tumor metastasis. *Biochim. Biophys. Acta*, **1032**, 89-118.

BLOOM HJ AND RICHARDSON WW. (1957). Histological grading and prognosis in breast cancer. *Br. J. Cancer*, **11**, 359-377.

BOSARI S, LEE AKC, DELELLIS RA, WILEY BD, HEATLEY GJ AND SILVERMAN ML. (1992). Microvessel quantitation and prognosis in breast carcinoma. *Human Pathol.*, **23**, 755-761.

FOLKMAN J. (1990). What is the evidence that tumors are angiogenesis dependent? *J. Natl Cancer Inst.*, **82**, 4-6.

FOLKMAN J. (1994). Angiogenesis and breast cancer. *J. Clin. Oncol.*, **12**, 441-443.

FOLKMAN J, COLE P AND ZIMMERMAN S. (1966). Tumor behavior in isolated perfused organs: *in vitro* growth and metastasis of biopsy material in rabbit thyroid and canine intestinal segment. *Ann. Surg.*, **164**, 491-502.

FOX SB, LEEK RD, SMITH K, HOLLYER J, GREENHALL M AND HARRIS AL. (1994). Tumour angiogenesis in node-negative breast carcinomas - relationship with epidermal growth receptor, estrogen receptor and survival. *Breast Cancer Res. Treat.*, **29**, 109-116.

GASPARINI G, WEIDNER N, BEVILACQUA P, MALUTA S, PALMA PD, CAFFO O, BARBARESCHI M, BORACCHI P, MARUBINI E AND POZZA F. (1994). Tumor microvessel density, p53 expression, tumor size, and peritumoral lymphatic vessel invasion are relevant prognostic markers in node-negative breast carcinoma. *J. Clin. Oncol.*, **12**, 454-466.

GOULD VE, LINNOILA LI, MEMOLI VA AND WARREN WH. (1983). Neuroendocrine components of the bronchopulmonary tract: hyperplasias, dysplasias, and neoplasms. *Lab. Invest.*, **49**, 519-537.

HALL NR, FISH DE, HUNT N, GOLDIN RD, GUILLOU PJ AND MONSON JR. (1992). Is the relationship between angiogenesis and metastasis in breast cancer real? *Surg. Oncol.*, **1**, 223-229.

HART IR AND SAINI A. (1992). Biology of tumour metastasis. *Lancet*, **339**, 1453-1457.

HORAK ER, LEEK R, KLENK N, LEJEUNE S, SMITH K, STUART N, GREENALL M, STEPNIEWSKA K AND HARRIS AL. (1992). Angiogenesis, assessed by platelet endothelial cell adhesion molecule antibodies, as indicator of node metastases and survival in breast cancer. *Lancet*, **340**, 1120-1124.

JENSEN HM, CHEN I, DEVAULT MR AND LEWIS AE. (1982). Angiogenesis induced by 'normal' human breast tissue: a probable marker for precancer. *Science*, **218**, 293-295.

LIOTTA LA, KLEINERMAN J AND SAIDEL GM. (1974). Quantitative relationships of intravascular tumor cells, tumor vessels, and pulmonary metastases following tumor implantation. *Cancer Res.*, **34**, 997-1004.

MACCHIARINI P, FONTANINI G, HARDIN MJ, SQUARTINI F AND ANGELETTI CA. (1992). Relation of neovasculature to metastasis of non-small-cell lung cancer. *Lancet*, **340**, 145-146.

MCGUIRE WL. (1989). Adjuvant therapy of node-negative breast cancer. *N. Engl. J. Med.*, **320**, 525-527.

MOSCATELLI D, GROSS J AND RIFKIN D. (1981). Angiogenic factors stimulate plasminogen activator and collagenase production by capillary endothelial cells. *J. Cell Biol.*, **91**, 201a.

OSBORNE CK. (1992). Prognostic factors for breast cancer: have they met their promise? *J. Clin. Oncol.*, **10**, 679-682.

SCARFF RW AND TORLONI H. (1968). *Histological Typing of Breast Tumors*. pp. 13-20. WHO: Geneva.

SIGURDSSON H, BALDETORP B, BORG A, DALBERG M, FERNO M, KILLANDER D AND OLSSON H. (1990). Indicators of prognosis in node-negative breast cancer. *N. Engl. J. Med.*, **322**, 1045-1053.

SRIVASTA A, LAIDLER P, DAVIES RP, HORAN K AND HUGHES LE. (1988). The prognostic significance of tumor vascularity in intermediate thickness (0.76-4.0 mm thick) skin melanoma: a quantitative histologic study. *Am. J. Pathol.*, **133**, 419-423.

SUTHERLAND RM, MCCREDIE JA AND INCH WR. (1971). Growth of multicell spheroids in tissue culture as a model of nodular carcinomas. *J. Natl Cancer Inst.*, **46**, 113-120.

TOI M, KASHITANI J AND TOMINAGA T. (1993). Tumor angiogenesis is an independent prognostic indicator in primary breast carcinoma. *Int. J. Cancer*, **55**, 371-374.

VAN HOEF MEHM, KNOX WF, DHESI SS, HOWELL A AND SCHOR AM. (1993). Assessment of tumor vascularity as a prognostic factor in lymph node negative invasive breast cancer. *Eur. J. Cancer*, **29A**, 1141-1145.

VISSCHER DW, SMILANETZ S, DROZDOWICZ S AND WYKES SM. (1993). Prognostic significance of image morphometric microvessel enumeration in breast carcinoma. *Anal. Quant. Cytol. Histol.*, **15**, 88-92.

WEIDNER N, SEMPLE JP, WELCH WR AND FOLKMAN J. (1991). Tumor angiogenesis and metastasis - correlation in invasive breast carcinoma. *N. Engl. J. Med.*, **324**, 1-8.

WEIDNER N, FOLKMAN J, POZZA F, BEVILACQUA P, ALLRED EN, MOORE DH, MELI S AND GASPARINI G. (1992). Tumor angiogenesis: a new significant and independent prognostic indicator in early-stage breast carcinoma. *J. Natl. Cancer Inst.*, **86**, 1875-1887.

WEIDNER N, CARROLL PR, FLAX J, BLUMENFELD W AND FOLKMAN J. (1993). Tumor angiogenesis correlates with metastasis in invasive prostate carcinoma. *Am. J. Pathol.*, **143**, 401-409.